# Sex differences in the skeletal muscle response to metformin treatment and the possible association with gut-muscle axis in rats

**DOI:** 10.3389/fendo.2025.1650805

**Published:** 2025-09-01

**Authors:** Lin Song, Rui Wang, Jiaqi Cui, Shuyuan Hu, Jingyue Wang, Jiaming Xie, Pudan Miao, Bo Sun

**Affiliations:** ^1^ Department of Physiology and Pathophysiology, School of Basic Medical Sciences, Xi’an Jiaotong University Health Science Center, Xi’an, Shaanxi, China; ^2^ Institute of Neuroscience, Translational Medicine Institute, Xi’an Jiaotong University Health Science Center, Xi’an, Shaanxi, China; ^3^ Department of Health Management, The First Affiliated Hospital of Xi’an Jiaotong University, Xi’an, Shaanxi, China; ^4^ Xi’an Jiaotong University Health Science Center, Xi'an, Shaanxi, China

**Keywords:** metformin, muscle, gut microbiota, short chain fatty acid, sex differences

## Abstract

**Introduction:**

Metformin (MT) is widely used in treating type 2 diabetes, and muscle is one of the targets for MT action. Recent studies have shown that gut microbiota plays a key role in mediating the clinical effects of MT, as well as affects muscle function, through the gut-muscle axis. However, it is not clear whether the use of MT in non-diabetic population affects muscle metabolism via gut-muscle axis and whether there are sex differences.

**Methods:**

We investigated the effects of ten days MT administration (200 mg/kg/d) on metabolic phenotype, skeletal muscle morphology and function-related gene expression, gut microbiota composition, gut integrity and inflammation, and plasma short chain fatty acids (SCFAs) levels in adult male and female Sprague-Dawley rats.

**Results:**

We found MT treatment decreased body weight, blood glucose and muscle gene expression involved in myogenesis and mitochondrial biogenesis and dynamics more significant in females, while increased the colonic mRNA expression of more inflammatory markers in males. MT treatment also induced sex-specific alterations in the gut microbiota composition, plasma SCFAs contents and muscle SCFA receptors’ mRNA expression in non-diabetic rats.

**Conclusions:**

Our research provides evidence that the use of MT in daily health maintenance may have sex-specific effects on gut-muscle axis and should be approached with caution.

## Introduction

Metformin (MT) is widely used as the first-line pharmacological treatment for type 2 diabetes mellitus. Beyond its approved indications, it has been reported to exert diverse pharmacological effects, including weight reduction and decreased food intake (1), protective actions against age-related diseases (2), enhancement of autophagy and mitochondrial function (3), antitumor activity (4), improvement of polycystic ovary syndrome (5), and potential benefits for cognitive function (6), among others. These pleiotropic properties have led some to propose that MT may represent a broad-spectrum therapeutic agent. Nevertheless, whether it is appropriate for routine use in daily health maintenance remains controversial.

Evidence supports the effectiveness of MT treatment for improving liver function and body composition in non-diabetic non-alcoholic fatty liver disease patients (7). A systematic review and meta-analysis suggest that MT use is associated with a decreased risk of overall cancer as well as several cancer subtypes, but with high heterogeneity and risk of population bias (8). Besides, ongoing human MT trials provided the first direct evidence that MT modulates metabolic and non-metabolic gene expression related to aging (9) and the protective effects of MT against several age-related diseases in humans will be tested in the Targeting Aging with Metformin trail (10). However, in non-diabetic patients with high cardiovascular risk, MT had little or no effect on several surrogate markers of cardiovascular disease (11). Further, not all individuals prescribed MT derive beneficial effects and some develop side effects. Thus, a more detailed understanding of the off-label uses of this drug is needed.

Muscle is one of the target organs for MT action, accounting for approximately 40% of total body weight, 30% of resting energy consumption and 80% of insulin stimulated glucose uptake (12). Muscle regulates multiple physiological functions, and losses of muscle mass and function can have negative impact on blood glucose control and create a vicious cycle with metabolic disorder. MT improves insulin sensitivity, enhances protein degradation and decreases blood glucose levels by activating AMP-activated protein kinase (AMPK) signaling pathway (13). Long-term MT treatment often accompanies weight loss, which is associated with a decrease in fat content (14). However, some studies report that MT administration might reduce lean mass content by inducing muscle atrophy in type 2 diabetes mellitus (T2D) patients (15, 16).

More and more new studies have confirmed that the clinical effects of MT are partly mediated by gut microbiota (17, 18). MT could alter the gut microbiota composition, thus affect the integrity of the intestinal barrier, regulate short-chain fatty acids (SCFAs) production and bile acid metabolism, and finally maintain homeostasis (18). Recent human studies suggest that MT could increase the abundance of Akkermansia muciniphila and several SCFA-producing microbiota, which produce butyrate, propionate, or substance involved in glucose homeostasis (19). The interaction between gut microbiota and host organs regulates the occurrence and development of various chronic diseases, such as obesity, diabetes, and autism, through mechanisms as gut-brain axis, gut-liver axis, etc (20, 21). In recent years, the cross-talk between gut microbiota and skeletal muscle (SM) has become a research hotspot, and the gut-muscle axis has been innovatively proposed, which means that the muscle function and metabolism largely depend on the quantity and composition of gut microbiota (22). Therefore, we aim to study whether the use of MT in the non-diabetic animal model affects SM metabolism and function, and whether MT treatment reshapes gut microbiota and its possible association with the changes in muscle.

In general, except for its well-known benefits, MT-associated adverse drug reactions (ADRs) are common, and women reported an ADR more often than men (23). Thus, apart from observing whether MT administration has beneficial effects on the metabolic phenotype, muscle function, gut integrity and inflammation, gut microbiota composition and plasma SCFAs levels in a rat model, we also intend to see whether there are sex differences when using MT for daily health maintenance in non-diabetic rats.

## Methods

### Animals

Adult male and female Sprague-Dawley rats were purchased from the Experimental Animal Center of Xi’an Jiaotong University. All rats were habituated individually for one week in a temperature- (22-24°C) and light- (light onset at 0800) controlled room, and had free access to standard lab chow (Beijing Ke Ao Xie Li, Beijing, China) and tap water. All animal experiments have been approved by the ethics committee of Xi’an Jiaotong University (No. 2022-1185) and strictly comply with the national regulations on the administration of experimental animals.

After habituation, 15 male and 16 female rats were randomized to receive metformin (MT, Sigma-Aldrich, St Louis, MO, USA) administration (200 mg/kg/d) in drinking water or normal drinking water (control group, CT) for ten days. All rats remained on their chow diet throughout the experiment. The body weight was measured every other day and the food intake was weighed daily.

### Sample collection

After 10 days MT treatment, the animals were fasted overnight and decapitated at 0900 (Male, CT, n = 7; MT, n = 8; female, CT, n = 8; MT, n = 8). Blood glucose was determined by a handheld glucose meter (ONETOUCH Ultra Vue, LifeScan, CA, USA). The subcutaneous (SC) fat and retroperitoneal (RP) fat were bilaterally dissected and weighed. The gastrocnemius tissue, colon tissue and colon content were quickly collected and snap-frozen in liquid nitrogen, and then stored at -80°C until analysis.

### Quantitative real-time PCR analysis

The qPCR was used to determine relative mRNA expression of genes related to the myogenesis (MyoD, MyoG, Myf5, Mrf4, Pax7 and Ctnnb1), mitochondrial biogenesis (Ppargc1a, Tfam, Nrf1) and dynamics (Fis1, Opa1, Drp1, Mfn1 and Mfn2), and SCFA receptors (Gpr41, Gpr109a) in the gastrocnemius tissue, and genes related to gut integrity (Tjp1, Ocln, Cldn4, and Muc2) and inflammatory status (Tnfa, Il1b, Il6, Il10, Cd3, Cd68, Hmgb1, Tlr2, Tlr4, Rage) in the colon tissue. Total RNA was isolated from muscle or colon homogenates using RNA isolation kit (R0027, Beyotime, Beijing, China). RNA was reverse transcribed to cDNA using Reverse transcription kit purchased from Thermo Scientific (K1622, MA, USA). Gene expression was determined by qPCR using SYBR green dye with specific primer sets in an iQ5 PCR thermal cycler (Bio-Rad, CA, USA). To determine the relative expression values, the −ΔΔCt method was used and normalized to the reference gene Actb. The primers of the genes studied can be found in our previous studies (24, 25).

### Histology of gastrocnemius

The gastrocnemius tissue was fixed in 4% paraformaldehyde for 24h, dehydrated and embedded in paraffin, then cut into 3 μm thick sections. The sections were stained with H&E and visualized under a light microscope (Olympus, Tokyo, Japan). The percentage of interstitium was evaluated in three different microscopic fields at 20× magnification of each muscle section and quantified using ImageJ software (NIH, MD, USA).

### Fecal microbiota composition

The fecal specimens were sent to GENEWIZ, Inc. (Suzhou, China) for 16S ribosomal DNA gene sequencing. The detailed process was described previously (24). Using VSEARCH clustering (1.9.6) program, sequences were clustered into operational taxonomic units (OTUs). Then use RDP classifier (Ribosomal Database Program) Bayesian algorithm of OTU species taxonomy analysis representative sequences, and under different species classification level statistics community composition of each sample. Values for alpha diversity (Chao1 Index, Shannon’ s Index), beta diversity (unweighted UniFrac distance metrics) and principal coordinate analysis (PCoA) plots were generated by QIIME V.1.9.1 based on the OTU analysis results.

### Detection of SCFAs

Plasma SCFAs contents were detected by MetWare (http://www.metware.cn/, Wuhan, China) based on the Agilent 8890-7000D GC-MS/MS platform.

### Statistical analysis

Statistical analysis between groups was analyzed using Student’s t-test or repeated measures analysis of variance with Prism 8 (GraphPad Software, CA, USA). All data are presented as the mean ± SEM, and statistical significance was set at *P* < 0.05.

## Results

### Effects of metformin treatment on metabolic phenotype of male and female rats

**Figure 1:**
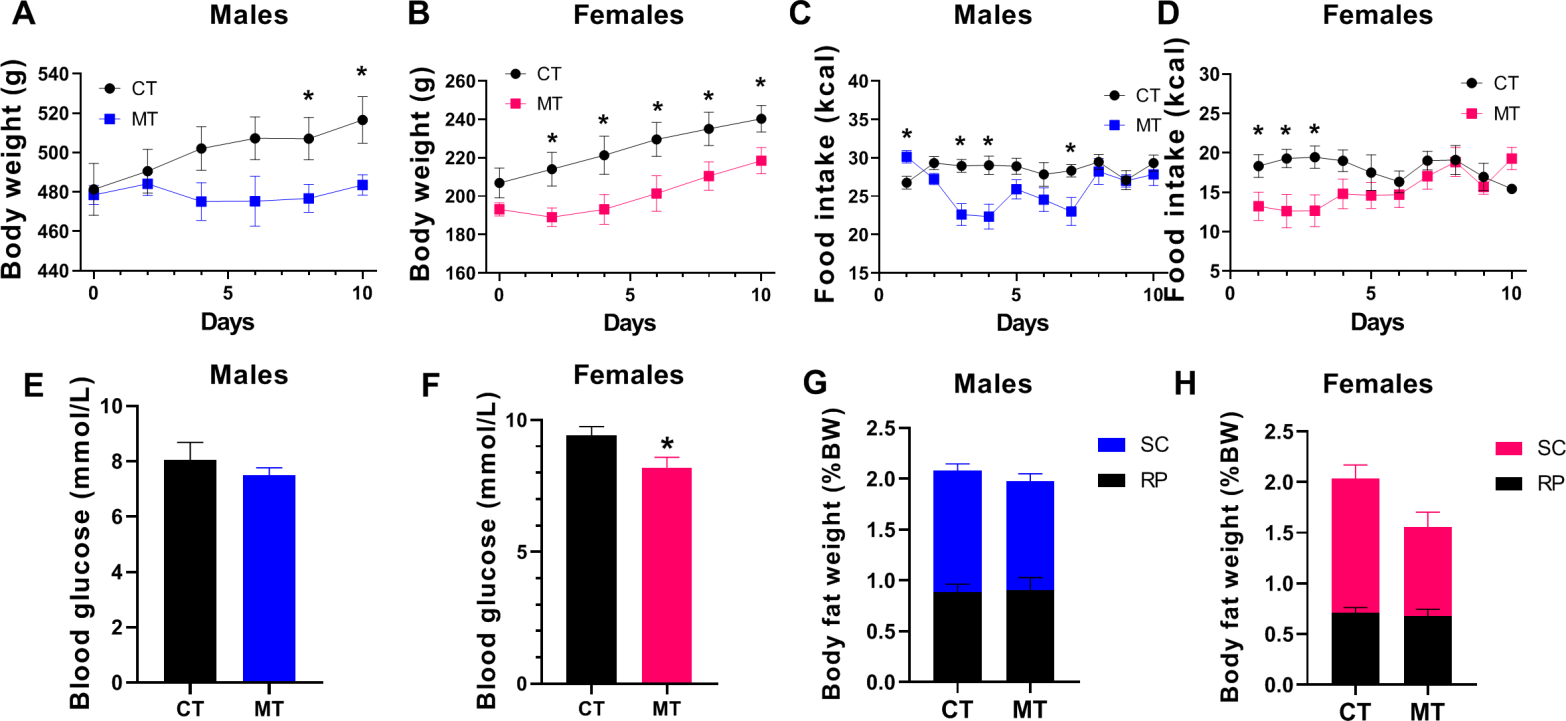
Effects of metformin treatment on metabolic phenotype of male and female rats. **(A, B)** Body weight of male and female rats during metformin (MT) treatment; **(C, D)** Food intake of male and female rats during MT treatment; **(E, F)** Blood glucose level at the end of MT treatment in male and female rats; **(G, H)** Male and female rats’ SC and RP fat weight (% body weight) at the end of MT treatment. Data are presented as the mean ± SEM. Male: CT, n = 7; MT, n = 8; Female: CT, n = 8; MT, n = 8. *P< 0.05, MT vs. CT.

In adult male rats, MT treatment started to decrease body weight on treatment day 8 and 10 (**Figure 1A**). However, in female rats, body weight was significantly reduced since treatment day 2 (**Figure 1B**). The food intake was decreased during the first week of MT treatment (except for day 1) in males (**Figure 1C**). In females, MT administration reduced food intake only during the first 3 days of treatment (**Figure 1D**). MT treatment reduced blood glucose in females but not in males (**Figures 1E, F**). Though both male and female rats had decreased body weight at the end of MT treatment, the SC and RP fat weight was not altered by MT treatment (**Figures 1G, H**).

### Effects of metformin treatment on mRNA expression of myogenesis, mitochondrial biogenesis and dynamics-related genes in skeletal muscle of male and female rats

**Figure 2:**
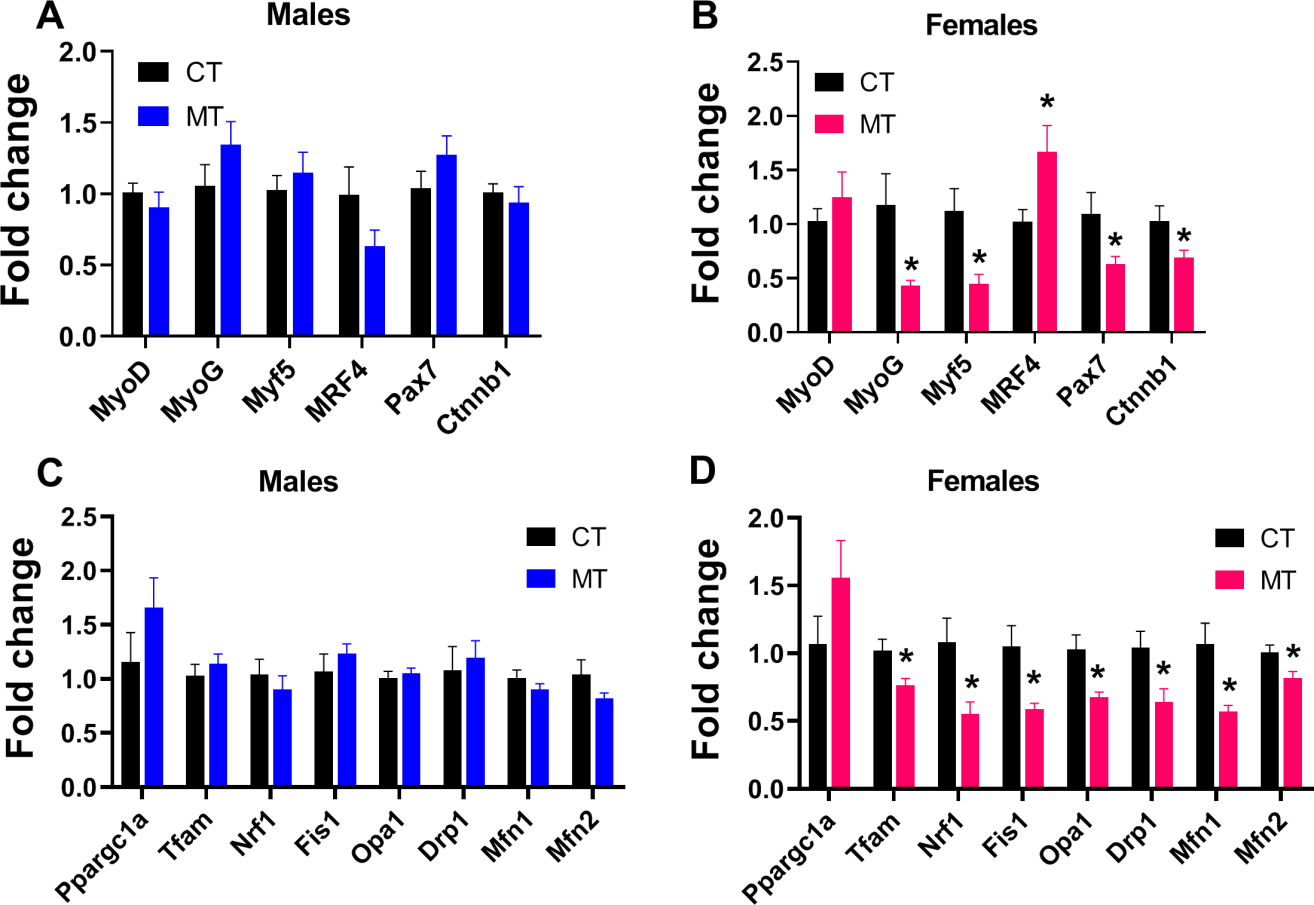
Effects of metformin treatment on gene expression involved in myogenesis, mitochondrial biogenesis and dynamics in skeletal muscle of male and female rats. **(A, B)** mRNA expression of myogenesis-related genes in skeletal muscle of male and female rats; **(C, D)** mRNA expression of mitochondrial biogenesis- and dynamics-related genes in skeletal muscle of male and female rats. Data are presented as the mean ± SEM. Male: CT, n = 6; MT, n = 6; Female: CT, n = 6; MT, n = 6. *P< 0.05, MT vs. CT.

We analyzed expression of genes in myogenic regulatory factors (MRFs) family including myogenic determining factor (MyoD), Myogenin (MyoG), myogenic factor 5 (Myf5) and MRF4, and genes regulate MRFs expression including the paired box transcription factor Pax7 and Ctnnb1 (encodes β-catenin). The expression of Pax7 and ctnnb1 controls muscle tissue differentiation and growth. In adult male rats, the mRNA expression of these myogenesis related genes was not altered by MT treatment (**Figure 2A**). However, MyoG, Myf5, Pax7 and Ctnnb1 gene expression was significantly decreased, while MRF4 gene expression was significantly increased by MT treatment in adult female rats (**Figure 2B**).

To investigate the effect of MT treatment on mitochondrial biogenesis and dynamics (fusion and fission) in SM, we determined mRNA expression of genes involved in these processes. In males, MT treatment did not affect mRNA expression of genes regulating mitochondrial biogenesis or dynamics (**Figure 2C**). To the contrary, MT treatment significantly reduced the mRNA expression of genes involved in mitochondrial biogenesis (Tfam, Nrf1) and dynamics (Fis1, Opa1, Drp1, Mfn1 and Mfn2) in adult female SM (**Figure 2D**).

### Effects of metformin treatment on morphology of skeletal muscle in male and female rats

**Figure 3:**
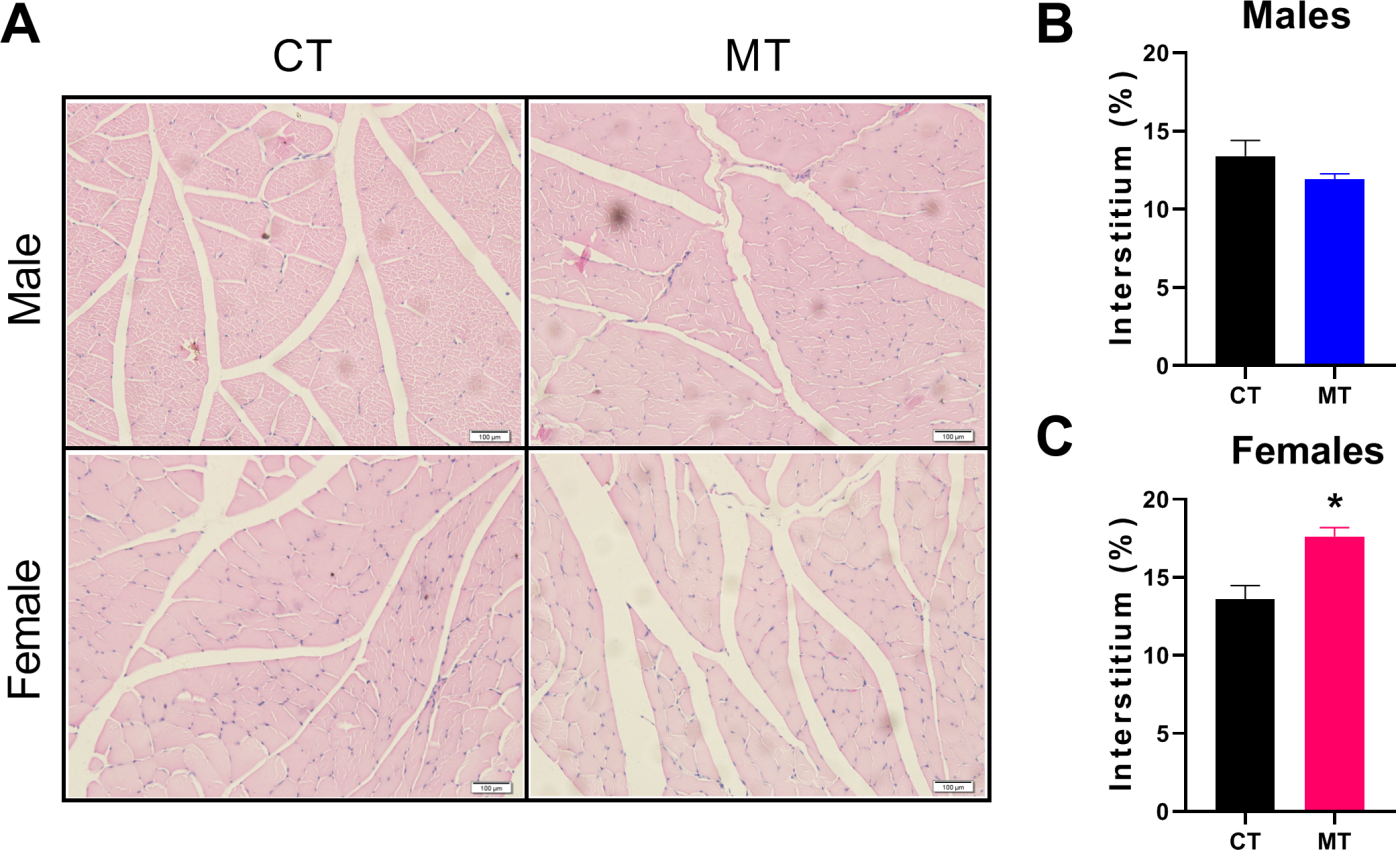
Effects of metformin treatment on morphology of skeletal muscle in male and female rats. **(A)** Representative skeletal muscle sections of H&E staining, Scale bar = 100 μm; **(B, C)** Percentage of interstitium in skeletal muscle of male and female rats. Data are presented as the mean ± SEM. Male: CT, n = 4; MT, n = 4; Female: CT, n = 3; MT, n = 5. *P< 0.05, MT vs. CT.

H&E staining was performed to show the morphology of skeletal muscle. We observed the percentage of interstitium in control and MT treated rats. In females, MT treatment significantly increased the percentage of interstitium compared with the CT group (**Figures 3A, C**). However, no significant differences were found between the CT and MT groups in males (**Figures 3A, B**).

### Effects of metformin treatment on gut microbiota composition in male and female rats

**Figure 4:**
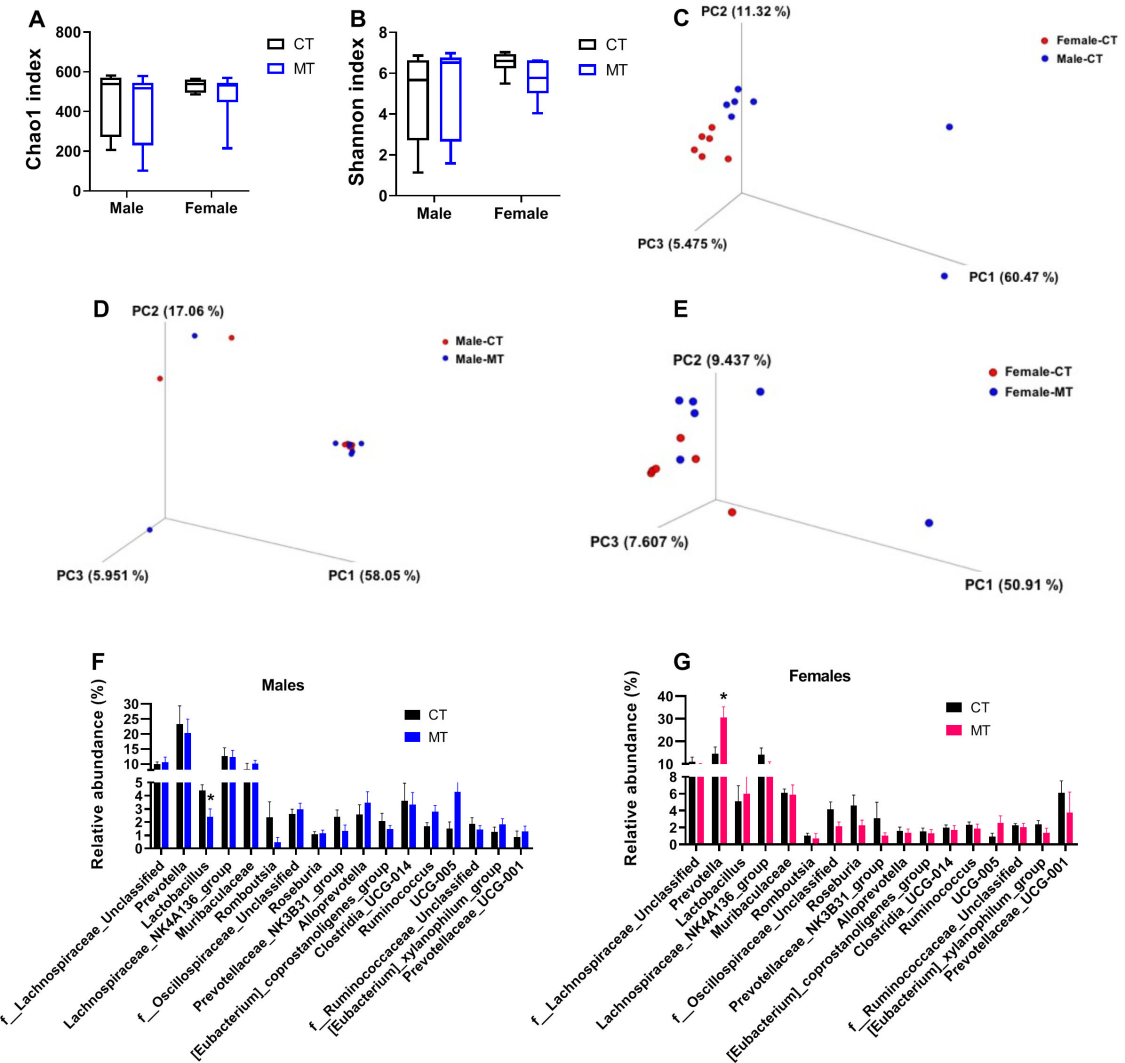
Effects of metformin treatment on gut microbiota composition in male and female rats. **(A, B)** Chao1 index and Shannon index for male and female rats; **(C)** PCoA 3D plot of weighted UniFrac distances in 16S rRNA sequencing of colonic contents in control (CT) male and female rats; **(D, E)** PCoA 3D plot of 16S rRNA sequencing of colonic contents in CT and metformin (MT) treated male and female rats; **(F, G)** Relative abundance of gut microbiota at genus levels in colonic contents of male and female rats. Each dot represents data from one rat; Male: CT, n = 7; MT, n = 7; Female: CT, n = 6; MT, n = 6. *P< 0.05, MT vs. CT.

Using 16S rDNA sequencing, we investigated the gut microbiota composition of male and female rats after treated with MT. Changes of alpha diversity parameters after MT treatment are presented as Chao1 index (community richness) and Shannon index (community diversity). We did not find any difference in alpha diversity between control and MT treatment group in either male or female rats (**Figures 4A, B**).

Beta diversity, which reflects species similarity, is presented in 3D PCoA charts in this study. As expected, a clear separation was observed in male and female rats in the control group (ANOSIM, P = 0.014, R = 0.292) (**Figure 4C**). In males, the dots representing the MT group were not significantly separated from the control group (ANOSIM, P = 0.367, R = 0.007) (**Figure 4D**). In females, significant separation between CT group and MT group was observed (ANOSIM, P = 0.047, R = 0.185) (**Figure 4E**).

Then, the relative abundance of fecal microbiota at genus level was analyzed in both male and female rats. We found that the relative abundance of *Lactobacillus* was significantly decreased in the MT group of male rats (**Figure 4F**). In females, the relative abundance of *Prevotella* was significantly upregulated by MT treatment (**Figure 4G**).

### Effects of metformin treatment on gut integrity and inflammatory conditions in male and female rats

**Figure 5:**
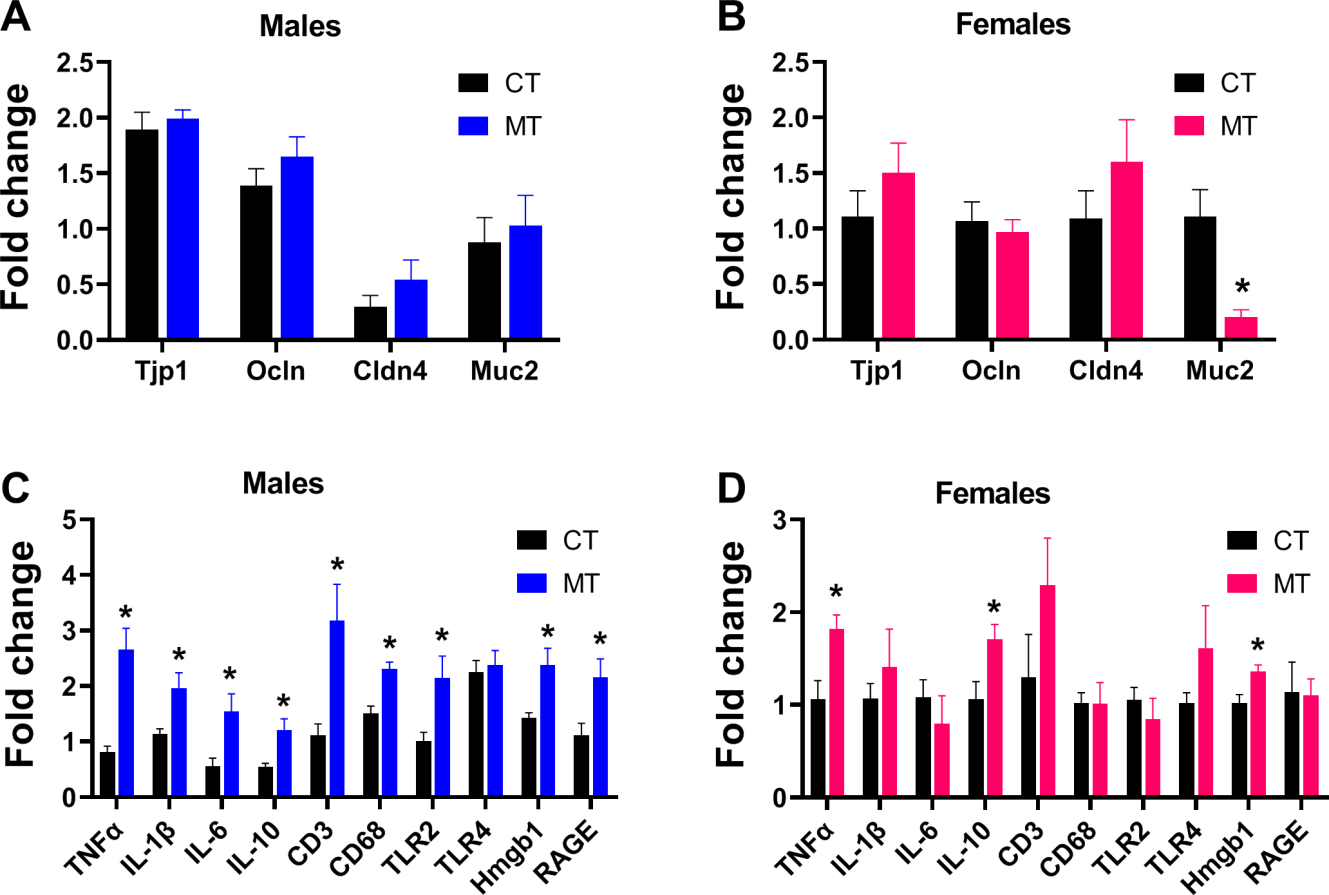
Effects of metformin treatment on gut integrity and inflammatory conditions in male and female rats. **(A, B)** Colonic mRNA expression of tight junction proteins in male and female rats; **(C, D)** Colonic mRNA expression of inflammatory markers in male and female rats. Data are presented as the mean ± SEM. Male: CT, n = 6; MT, n = 6; Female: CT, n = 6; MT, n = 6. *P< 0.05, MT vs. CT.

Reshaped gut microbiota composition is known to be associated with gut barrier dysfunction and gut inflammation. We investigated if MT treatment affected gut integrity by determining the mRNA expression of the tight junction protein 1 (Tjp1), occludin (Ocln), claudin 4 (Cldn4) and mucin 2 (Muc2). In males, MT treatment had no effect on the gene expression of tight junction markers in the colon tissue (**Figure 5A**). However, in females, MT treatment significantly reduced gene expression of Muc2 in colon (**Figure 5B**).

To investigate the gut inflammatory status, colonic gene expression of inflammation markers (TNFα, IL-1β, IL-6, IL-10, CD3, CD68, TLR2, TLR4, Hmgb1 and RAGE) was measured. MT treatment significantly increased the mRNA expression of TNFα, IL-1β, IL-6, IL-10, CD3, CD68, TLR2, Hmgb1 and RAGE in male rats (**Figure 5C**), while increased the mRNA expression of TNFα, IL-10 and Hmgb1 in the females (**Figure 5D**).

### Effects of metformin treatment on plasma SCFAs levels and mRNA expression of SCFA receptors in skeletal muscle of male and female rats

**Figure 6:**
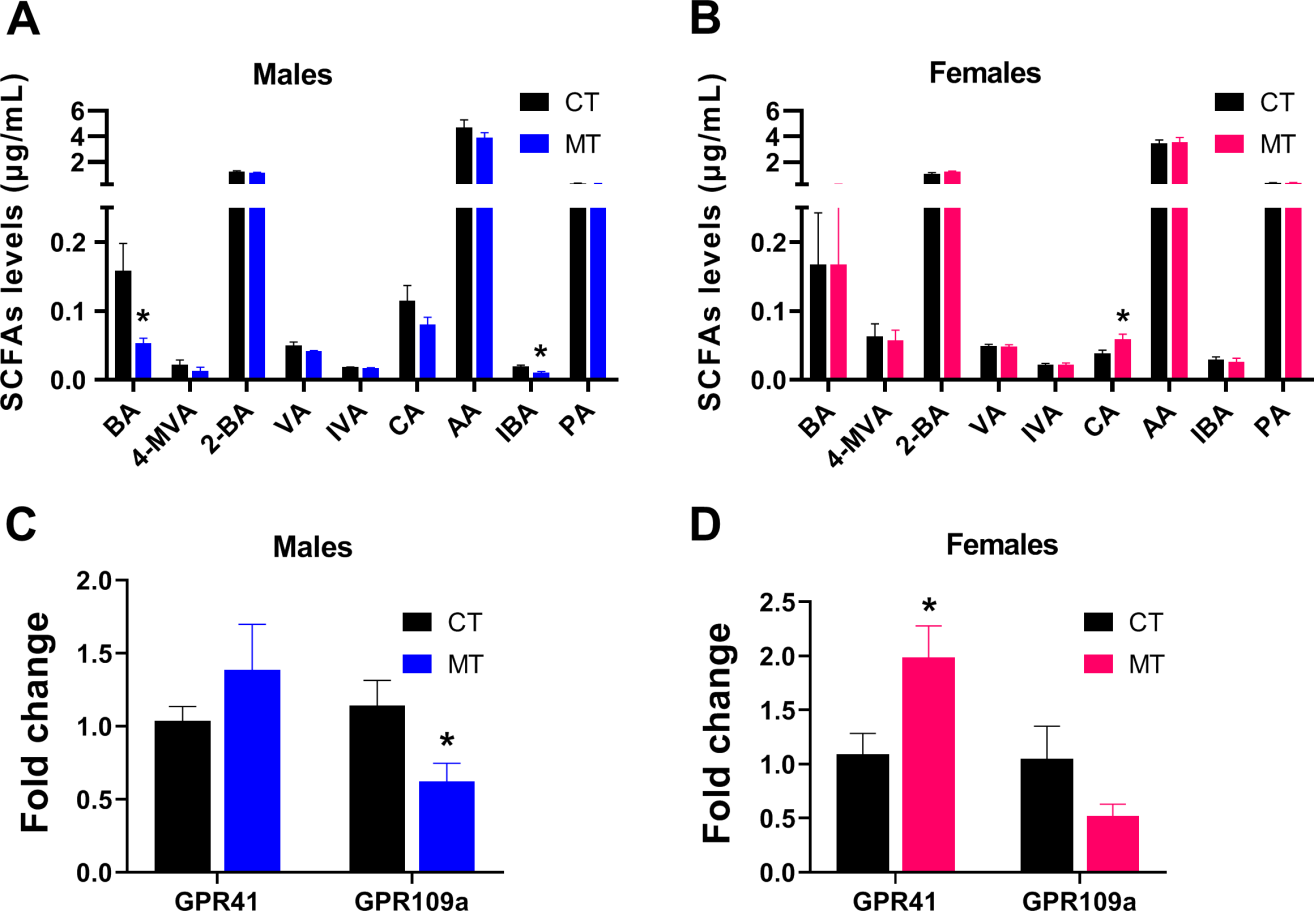
Effects of metformin treatment on plasma SCFAs levels and mRNA expression of SCFA receptors in skeletal muscle of male and female rats. **(A, B)** Plasma SCFAs levels in male and female rats; **(C, D)** Gene expression of SCFA receptors in skeletal muscle of male and female rats. Data are presented as the mean ± SEM. Male: CT, n = 6; MT, n = 6; Female: CT, n = 6; MT, n = 6. *P< 0.05, MT vs. CT. BA, butyric acid; 4-MVA, isocaproic acid; 2-BA, 2-methylbutyric acid; VA, valeric acid; IVA, isovaleric acid; CA, caproic acid; AA, acetic acid; IBA, isobutyric acid; PA, propionic acid.

SCFAs are produced mainly through interaction between diet and the gut microbiota. We found the plasma levels of butyric acid (BA) and isobutyric acid (IBA) were significantly reduced in male MT treated rats (**Figure 6A**). However, the plasma level of caproic acid (CA) was significantly increased in female MT treated rats (**Figure 6B**).

Then, we analyzed the mRNA expression of SCFA receptors in SM. In male rats, mRNA expression of GPR109a was significantly decreased after MT treatment (**Figure 6C**). In female rats, mRNA expression of GPR41 was significantly increased after MT treatment (**Figure 6D**).

## Discussion

Large cohort studies have shown the weight loss benefits of MT, especially in the Diabetes Prevention Program (DPP) (26). The DPP showed that high-risk participants have reduced incidence of diabetes by 31% in a 3-year period when treated with MT, and the weight loss associated with MT was safe and sustained (27). Initial studies suggest that the weight change associated with MT is due to its impact on hypothalamic appetite regulatory centers (28). However, a randomized controlled trail of 4.3 year suggests that the prevention of weight gain by MT cannot be explained by reduced energy intake (29). Consistent in our study, the MT-associated weight loss was not relevant to food intake, and the body fat content was not significantly altered by MT treatment, so we speculate that the reduced body weight may relate to loss of lean mass, especially in MT-treated female rats. Kang et al. also found that MT induces muscle atrophy, and the muscle-wasting effect of MT is more evident in wild-type mice than in db/db mice (16).

Currently, the impact of MT on muscle is controversial. The blood glucose level is inversely associated with muscle mass in a healthy population (30). In this study, female MT-treated rats had lower blood glucose levels, however, the gene expression of myogenesis markers (MyoG, Myf5, Pax7 and Ctnnb1) was significantly reduced. MRF4 represses the activity of myocyte enhancer factor 2, negatively regulates adult skeletal muscle growth (31), whose expression was significantly increased in our study. The Pax7 gene is an identity marker for muscle satellite cells, and decreased Pax7 expression indicates exhaustion of the stem cell pool, leading to loss of self-renewal and regenerative capacity (32). The Ctnnb1 gene encodes the β-catenin protein, which is a core effector of the Wnt signaling pathway. The decreased Ctnnb1 expression suggests inhibition of the Wnt/β-catenin signaling pathway, which implies reduced pro-proliferative signaling and attenuated driving forces for fibrosis (33). Besides, MT treatment also led to muscle remodeling by increasing extramyocyte space in female rats, which may cause skeletal muscle dysfunction (34). Combined the results of increased proportion of the interstitium in female muscle fibers, MT administration may cause the muscle enters a “silent atrophy” state with regenerative exhaustion and no fibrosis driver. Predominantly, MT improves insulin resistance and decrease hepatic glucose production through activation of AMPK signaling pathway (35). The activation of AMPK signaling inhibits anabolic processes such as protein syntheses, promotes protein degradation and autophagy (36). Kang et al. suggests that MT induces muscle atrophy through AMPK-HDAC6-FoxO3a-Myostatin axis (16) by inhibiting the expression of MyoD and MyoG (37), which supports our results. However, several human studies found that MT treatment did not change fat-free mass significantly (38, 39) or even increased the lean mass and water content (40). One of the primary targets of MT in muscle is mitochondria (41). In the current study, we found MT treatment significantly reduced the expression of transcriptional factors involved in mitochondrial biogenesis (Nrf1 and Tfam) and mitochondrial membrane fusion (Mnf1, Mnf2, and Opa1) and fission (Fis1 and Drp1) in female rats, suggesting impaired biogenesis and quality of the mitochondria, and reduced energy metabolism efficiency. Several *in vitro* studies have shown that MT inhibits Complex I of the mitochondrial respiratory chain (42, 43), and one *in vivo* study reports that two weeks of MT treatment impairs muscle oxidative capacity in a dose-dependent manner (41).

Within the past few years, the close association between gut microbiota and SM has been revealed and accumulating evidences demonstrate that alteration in gut microbiota coincides with alteration in SM metabolism (44, 45). Preclinical studies have demonstrated that MT alters the gut microbiota composition and function (46, 47). In this study, we attempt to explain the impact of MT on muscle from the perspective of the 'gut-muscle axis'. Interestingly, MT treatment altered the gut microbiota composition significantly in females, but not in males, which coincides with the alterations in muscle. Specifically, MT treatment decreased the relative abundance of *Lactobacillus* in males, while increased the relative abundance of *Prevotella* in females. In contrary to our study, most other research found that MT treatment is associated with a significant increase in the abundance of *Lactobacillus* in male rodents (48, 49). However, Silamikele et al. demonstrates that the abundance of *Lactobacillus* was mainly reduced in response to long-term MT treatment in males (50). Several studies suggest that enriched *Lactobacillus* is associated with improved inflammation (51, 52). Consist with our study, the MT treated male rats had a deficiency of *Lactobacillus* and aggravated inflammatory conditions in colon. One study testing long-term MT treatment on the gut microbiome in non-diabetic status found that, microbes from the *Prevotellaceae* classes were enriched (46). However, one human study found an increased abundance of *Prevotella copri* in T2D patients who were not respond well to MT treatment (53), while another study has shown that *Prevotella* was enriched in gestational diabetes patients (54). *Prevotella* is involved in mucin oligosaccharide degradation and may impair gut permeability (55). Cuesta-Zuluaga et al. found that MT is associated with higher levels of SCFA-producing and mucin-degrading microbiota (56). Interestingly, we found decreased Muc2 mRNA expression in the colon of MT-treated female rats, which is coincided with the increased abundance of *Prevotella*.

The gut microbiota affects metabolic phenotype by fermenting indigestible dietary components and thereby producing SCFAs (57). Currently, SCFAs have been widely reported to improve SM function. For instance, supplementing SCFAs mixture can improve muscle atrophy and function of germ-free mice (58), while continuous subcutaneous injection of acetic acid can restore the endurance performance of antibiotic-treated mice (59). In this study, plasma BA levels were significantly reduced, together with increased inflammation in colon and decreased gene expression of GPR109a in the muscle of MT treated male rats. *Lactobacillus* produces lactate, which can increase BA production in feces and butyrate uptake in intestinal epithelial cells, and then promote gut hormone secretion and colonic integrity, as well as inhibit inflammation (60). Thananimit et al. report that selected probiotic *Lactobacillus* strains such as *L. paracasei* SD1 and *L. rhamnosus* SD11 could produce SCFAs, particularly butyrate (61). BA can bind to GPR109a and improve inflammation in gut and SM via regulation of NF-κB signaling (62, 63). Consistent with our results, MT treated male rats had reduced *Lactobacillus* abundance and worse inflammatory condition in colon. *Prevotella* showed high fiber-utilizing capacity and high production of total SCFAs with propionate as the major product (64). *Prevotella* is associated with glycan degradation, which can provide carbon sources to CA producing bacteria (such as the genus *Caproiciproducens*) (65). The female MT treated rats had increased *Prevotella* abundance and plasma CA levels, as well as higher GPR41 mRNA expression in SM in this study. A rodent study showed that increased acetic acid, propionic acid, CA and total SCFA levels activated the SCFAs-GPR41 pathway and the downstream mitogen-activated protein kinase signaling pathway (66). The activation of GPR41 by SCFAs can raise energy expenditure and may activate muscle energy catabolism (67), which may explain the impaired the myogenesis and mitochondrial function in muscle.

Although interesting and important discoveries were revealed by this study, some limitations still exist. This study mainly focused on the alterations MT treatment induced in muscle at gene expression level, the mechanism of SCFAs bind to its receptors and activate the downstream signaling to affect muscle function should be proved with additional experiments using gene-edited mice or SM cell lines. Further analysis with metagenomic sequencing will help to screen out specific strains changed in the intestinal flora after the action of MT, and will complete the chain of evidence that how gut-muscle axis functions in this study.

?>It is intriguing to find that SM responds to MT treatment in a sex-specific manner. Previous studies report that sex differences were found in the SM when response to high-fat/high sucrose diet or calorie restriction in rats (68, 69). First, the morphometric properties of muscle fibers show gender differences, such as the numbers, diameters, and cross-section areas of muscle fibers (70). A current meta-analysis revealed that distribution percentage of Type I muscle fiber are greater in women than men, whereas distribution percentage of Type II muscle fibers tend to be greater in men than women, which may contribute to sex differences in physical activity patterns (71). Second, mitochondrial dynamics and biogenesis are shaped by sex, with females had more functional mitochondrial than males in SM (72). SM from female rats showed higher mitochondrial DNA and protein contents, and oxidative-phosphorylative capacities than males (73). Furthermore, we found the gut microbiota composition was significant different between male and female rats. And Shi et al. suggest that sex determines gut microbes and their metabolites (SCFAs/MCFAs) in high fat diet fed rats (74). Sex disparities were also observed in treatment transitions after MT initiation among T2D patients (75). In juvenile mice, circulating adiponectin and insulin levels were altered by MT treatment in a sex-specific manner (76). Our results suggest that in healthy rats, short term MT treatment is more likely to lead to muscle atrophy in female, while cause worse gut inflammation in male.

**Figure 7:**
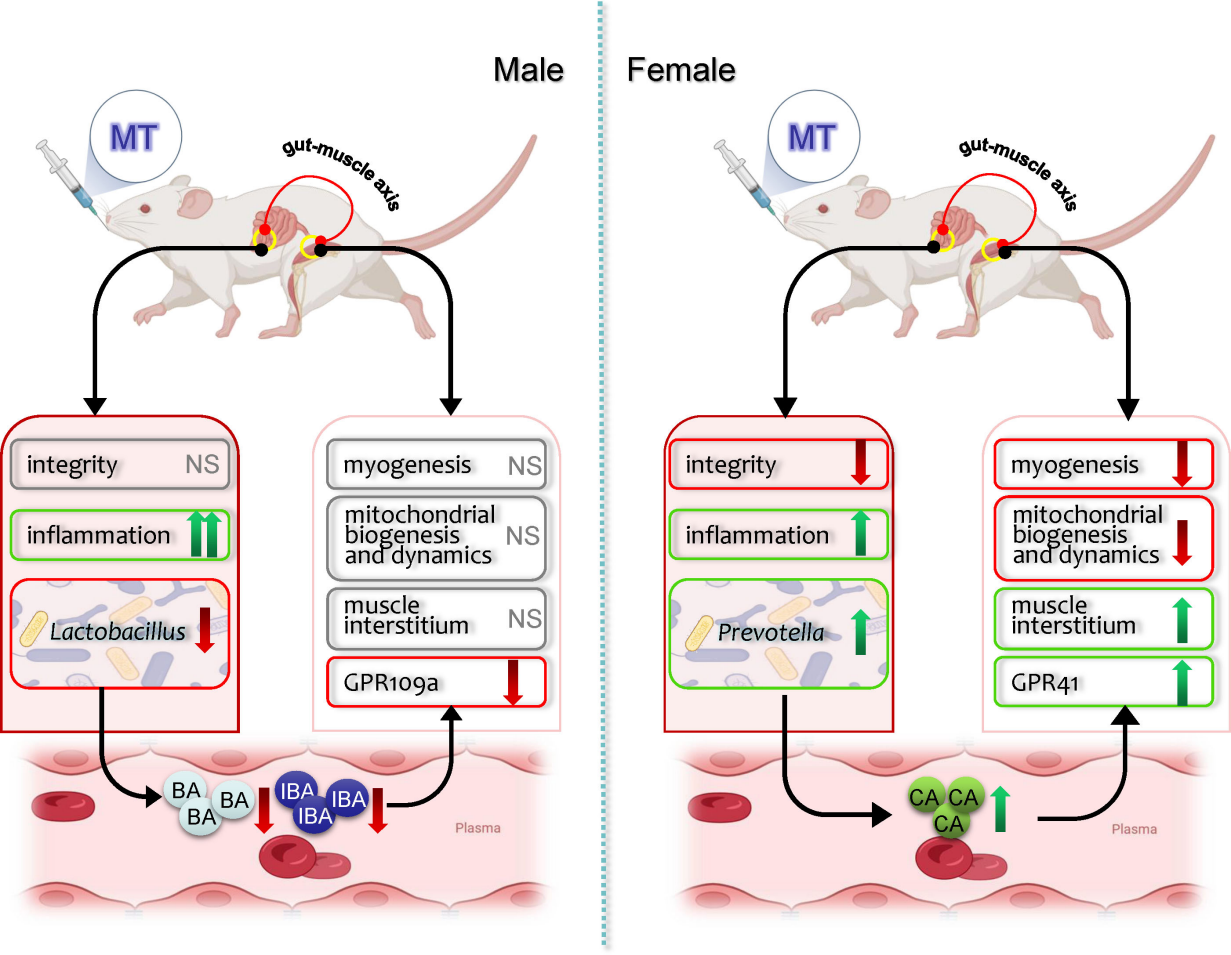
Schematic mechanism of metformin effects on muscle and gut microbiota in male and female rats. The up (↑) and down (↓) arrows indicate increased or decreased of its gene expression, relative abundance or plasma levels, respectively. MT, metformin; NS, no significant effects.

In conclusion, SM responses to MT treatment in a sexually dimorphic manner, which may partly associate with the gut-muscle axis (**Figure 7**). MT treatment decreased body weight, blood glucose and SM gene expression involved in myogenesis and mitochondrial function more significant in females, while increased the mRNA expression levels of more inflammatory markers in the gut of males. And MT treatment induced sex-specific alterations in the gut microbiota composition and plasma SCFAs contents in non-diabetic rats. Our research provides evidence that the use of MT in daily health maintenance has sex-specific effects on gut-muscle axis and should be approached with caution.

## Data Availability

The original contributions presented in the study are included in the article/supplementary material, further inquiries can be directed to the corresponding author/s.
